# Screening Method for Assessment of Work Ability for Patients Suffering From Mental Fatigue

**DOI:** 10.3389/fnbeh.2022.869377

**Published:** 2022-06-14

**Authors:** Birgitta Johansson

**Affiliations:** Institute of Neuroscience and Physiology, University of Gothenburg, Gothenburg, Sweden

**Keywords:** mental fatigue, work ability, TBI, stroke, hypothyriodism, burn-out

## Abstract

Pathological mental fatigue adversely affects endurance in the performance of tasks over time, with negative impact on work ability. There are currently no methods for objectively assessing work ability for patients suffering from mental fatigue. In this study, work ability in relation to mental fatigue as a screening method was evaluated, using the Work Ability Index (WAI) and Mental Fatigue Scale (MFS). Included participants represented three patient groups commonly affected by mental fatigue; acquired brain injury (*n* = 49, traumatic brain injury, stroke), burn-out syndrome (55) and hypothyroidism (50). The results showed a significant correlation between WAI and MFS (*r* = –0.754) and status in the workplace (WAI *r* = 0.722, MFS *r* = –0.443) for all groups. The WAI and MFS were significant predictors for status in the workplace (*p* < 0.001) and explained 53% of the variance. This screening method can help health care professionals to identify people who are mentally fatigued with a reduced ability to work or return to work after illness, and who are in need of in-depth investigation. It is important to initiate treatment early with the aim of promoting a sustainable working life and general well-being of the individual.

## Introduction

Long-lasting mental fatigue is a pathological state with an extreme mental energy deficit and reduced ability to regain this energy in response to activity, while “normal” fatigue is time-limited and alleviated by rest (Rönnbäck and Johansson, 2022). Pathological mental fatigue results in reduced quality of life and impaired ability to maintain employment or educational status after an acquired brain injury, endocrine or burn-out syndrome (Cantor et al., 2008; Glise et al., 2010; Johansson and Rönnbäck, 2014b; Palm et al., 2017; Leso et al., 2020). Work ability defined by Tengland (2011) is having the health status to perform work with tasks that are reasonable in an acceptable work environment. The so-called invisible mental energy deficit is difficult to conceptualize particularly in relation to work ability. From clinical experience, patients usually have a strong wish to return to ordinary life, including work, but they may not always understand or report their reduced capacity to work. If adequate support is missing, some will return to work and increase their working hours too quickly, with an increased risk of becoming more fatigued followed by a reduced work ability. Others may seem to lack the motivation or appear to be lazy. As many as 30% of family members and friends interpret fatigue after traumatic brain injury (TBI) as laziness (Norrie et al., 2010). This shows how difficult it can be to both understand mental fatigue and assess the patient’s work capacity, even for health professionals, if there is no team trained in assessing work ability in relation to mental fatigue.

Patients included in this study, commonly affected by long-term mental fatigue are those who have suffered an acquired brain injury (ABI, here TBI or stroke), been diagnosed with burn-out syndrome (BO) or hypothyroidism (HYT). Studies reporting work ability for these patient groups in relation to fatigue show that work ability can be reduced during many years and return to work can be difficult.

### Acquired Brain Injury Fatigue and Work Ability

From a systematic review, return to work improved with time, from 41% during the first 6 months, 53% at 1 year, to 66% 2–4 years after the stroke (Edwards et al., 2018). In a study, 2 years after a stroke, 58% of patients had returned to paid work (full-time or part-time), and higher fatigue scores (Multidimensional Fatigue Inventory-20, MFI) were associated with a reduced ability to return to paid work (Andersen et al., 2012). Fatigue (Fatigue Severity Scale, FSS) was associated with a return to work up to 12 months after discharge from rehabilitation and was not related to severity of stroke, age, cognitive impairment or depression. Fatigue was, according to the authors suggested to be routinely screened for and patients and employers informed about the impact that fatigue will have on a return to work (Rutkowski et al., 2021). Fatigue (FSS and Mental Fatigue Scale MFS) lasting 1–7 years after an aneurysmal subarachnoid hemorrhage was related to return to work. Among those employed before the hemorrhage, 55% had not returned to work, 35% on part time and 10% to full time work (Western et al., 2021). Fatigue (FSS, MFS) was a common complaint among people who had returned to work after a stroke where two-thirds of the women and half of the men reported that fatigue interfered with everyday life (Norlander et al., 2021). From a qualitative study, patients who had suffered a stroke and returned to work commonly reported fatigue, concentration and memory problems and personal change as having an impact on work ability (Balasooriya-Smeekens et al., 2016). One year after a TBI, fatigue (MFI) was associated with a lower status in the workplace (Beaulieu-Bonneau and Ouellet, 2017). Higher rating on fatigue (MFS) irrespective of TBI severity was associated with decreased work status in the workplace (Palm et al., 2017). After a mild TBI, fatigue (Barrow Neurological Institute Fatigue Scale) was a predictor for a slower return to work (Waljas et al., 2014).

### Hypothyroidism Fatigue and Work Ability

Fatigue is commonly reported in patients with autoimmune hypothyroidism (HYT) and these patients scored significantly higher than controls on all five MFI-20 subscales, this being independent of clinical and thyroid hormone parameters (Louwerens et al., 2012). It is less frequently reported of work ability in relation to fatigue among patients suffering from hypothyroidism, compared to acquired brain injury. However, from a review, thyroid diseases are reported as having an impact on work ability (Leso et al., 2020).

### Burn-Out Syndrome Fatigue and Work Ability

More straightforward, work ability can be related to mental health and energy depletion, and this is defined as the burn-out syndrome (BO) classified as an occupational phenomenon and not as a medical condition in ICD-11. BO is defined according to the dimensions: feelings of energy depletion or exhaustion; increased mental distancing from the person’s job; feelings of negativism or cynicism related to one’s job; and reduced professional efficacy. In Sweden, before the BO syndrome was classified, and with the need to define patients with fatigue/exhaustion due to long-term stress, the Exhaustion disorder was defined by the Swedish National Board of Health and Welfare (F43.8A). Exhaustion disorder is related to external identifiable loads such as psychosocial stress at work or in private life, or a combination of both, with a duration of 6 months or more, and including the central symptoms; lack of energy, disturbed sleep and cognitive problems (Åsberg et al., 2003). BO is a common cause of sick leave, and return to work can take many years and some may not resume work ability. From a 7-year follow-up study in Sweden, 3% were on full time sick leave, 4% had received sickness pension, 6% were on part time sick leave and 87% were not on sick leave (Beno et al., 2021).

From the studies referred to above, fatigue and work ability are related and show the importance of improving awareness and the need to assess mental fatigue in relation to work ability.

The purpose of this study is to evaluate a screening method for assessing work ability in three patient groups where mental fatigue is common. The patient groups included acquired brain injury (ABI including TBI and stroke), burn-out syndrome (BO) and hypothyroidism (HYT). This screening method can help health care professionals to identify people who suffer from mental fatigue with a reduced ability to work and who are in need of in-depth investigation.

## Materials and Methods

Twenty-three health care centers specializing in primary care in Gothenburg were contacted, and 15 of these consented to participate. Included patients have had contact with their health center during the past 5 years for acquired brain injury (ABI, TBI and stroke, diagnostic codes ICD-10: S06, I60, I63.0-9), hypothyroidism (HYT, deficiency of thyroid hormone, E03.9), and burn-out syndrome (BO, Swedish definition, Exhaustion disorder, F43.8A) (Åsberg et al., 2003). A randomized selection of participants was made by the Department of Data Management and Analysis, Region Västra Götaland, with 200 participants/group. Only one diagnosis was used for the selection of patients. The ABI group was prioritized for the selection, as this was the group with the least number of patients, second priority BO and lastly HYT as being the largest group. The ambition was to capture those who had recovered and those who were still struggling with mental fatigue in relation to work ability. A mail was sent to all the participants providing information concerning the study, the questionnaires, copy of a letter of approval from the head of the health center and a stamped addressed envelope. The study was approved by the Swedish Ethical Review Authority (2019-05177) and the Department of Data Management and Analysis, Region Västra Götaland (202-03789).

### Assessment

Participants were asked to fill in a form providing the following background information: age, education, actual percentage of full-time working hours (status in the workplace, full-time working hours 100%, or 75%, 50%, 25% or 0% of full-time payed work), to what extent they themselves perceived their work status (100%, or 75%, 50%, 25% or 0% of full-time payed work). They also reported whether they had an additional diagnosis of the three included in the study, as due to the selection procedure, some may have suffered from more than one of the included diagnoses. The questionnaires answered were Work Ability Index, WAI (de Zwar et al., 2002; van den Berg et al., 2009) and MFS (Johansson et al., 2010; Johansson and Rönnbäck, 2014a). The WAI has been used to assess individuals’ work ability and personal resources in relation to work requirements (Lundin et al., 2107). The WAI has good reliability and validity (de Zwar et al., 2002). The WAI consists of a questionnaire with 10 questions concerning the individual’s own physical and mental health, requirements at their place of employment in relation to their work ability, sick leave taken during the past year and whether the state of health will allow for continued work in the current profession 2 years ahead. The answers to the questions included in the questionnaire are given numerical values and are weighted together, according to a given formula to an index value (range 7–49) that indicates work ability; Poor (7–27), Moderate (28–36), Good (37–43) and Excellent (44–49) (de Zwar et al., 2002). Questionnaires and automatic calculations are available free of charge on the Internet. The Mental Fatigue Scale (MFS) has been evaluated for people with acquired brain injury (Johansson et al., 2010; Johansson and Rönnbäck, 2014a). The MFS has also been used in neurological conditions (Johansson et al., 2010; Bergqvist et al., 2019), endocrine diseases (Papakokkinou et al., 2015; Holmberg et al., 2021) and BO (Skau et al., 2021) and has also shown good correlation with status in the workplace after TBI (Palm et al., 2017). The MFS is a self-assessment form based on extensive clinical research into diseases that affect the brain (Lindqvist and Malmgren, 1993). A value above 10 indicates problems with mental fatigue (range 0–42). The cutoff score is calculated and a significant score of 10.5 was found to deviate significantly from the control sample (Johansson and Rönnbäck, 2014a). The higher the value, the greater the problems. The including questions cover topics concerning; generalized fatigue, mental fatigue, mental recovery, concentration and memory, slowness of thinking, stress sensitivity, sensitivity and irritability, initiative, light and sound sensitivity and sleep problems. The questions have a high internal consistency (Johansson et al., 2009). MFS is available free of charge on the Internet.

### Statistics

Chi-squared test was used to compare gender frequency, education, work status, and self-perceived work status. Analysis of variance (ANOVA) was used to compare basic data for the three included groups (age, MFS, WAI). Correlation of data was done with Pearson’s correlation and linear regression to test a model for variables’ predictive value for work ability. Statistical analyses were performed using SPSS-28.

## Results

A total of 154 people (26%) responded and 2% of the letters were returned without having been responded to out of a total of 600. The groups were similar in number of respondents, age and education. The ABI group had an even gender distribution, but significantly more women responded in the HYT and BO groups. Work status did not differ between groups, but the self-perceived work status differed significantly between groups (Table 1).

**TABLE 1:** Number of respondents (total 154), frequency, means, standard deviation (sd), statistical comparison between the ABI (acquired brain injury), HYT (hypothyroidism), and the BO (burn-out syndrome) (*p*-value) groups.

	ABI	HYT	BO	*p*-value
Number of respondents	49	50	55	
Age, mean in years (sd)	48 (13)	50 (13)	44 (12)	0.057^b^
Gender (women/men/non-binary)	28/21/1	39/10/1	43/12/0	0.025^a^
Education (elementary/high school/university)	5/20/24	4/15/31	6/16/33	0.759^a^
Status in the workplace, mean% (sd)	68 (41)	80 (34)	63 (43)	0.220^a^
Self-perceived work status, mean% (sd)	60 (36)	75 (28)	51 (33)	0.040^a^
Other self-reported diagnoses	3 HYT, 5 BO	1 ABI, 3 BO	4 ABI, 3 HYT	

*^a^Chi-squared test.*

*^b^ANOVA.*

A majority of the respondents had a reduced work ability as indicated with WAI scores and problems with mental fatigue as indicated with MFS. The BO group reported the lowest/worst WAI score and the highest MFS rating; the HYT group, the highest/best WAI score and least problems with mental fatigue and the ABI group reported scores in-between these (Table 2). The gender distribution differed between groups. The within-group comparison (t-test/group) did not find any difference between men and women of their MFS rating (ABI *p* = 0.594, HYT *p* = 0.683. BO *p* = 0.691) or WAI rating (ABI *p* = 0.715, HYT *p* = 0.767. BO *p* = 0.603).

**TABLE 2:** The WAI (Work Ability Index), the MFS (Mental Fatigue Scale) ratings for the ABI (acquired brain injury), HYT (hypothyroidism) and BO (burn-out syndrome) groups, and ratings for women and men, respectively. Mean, standard deviation and *p*-value.

	ABI	HYT	BO	*p*-value
MFS all	15.4 (7.5)	11.5 (7.5)	21.0 (5.9)	<0.001*
MFS women	15.4 (8.1)	12.3 (7.2)	22.0 (5.8)	
MFS men	15.5 (7.1)	8.5 (8.2)	17.6 (5.3)	
MFS numbers above 10	34 (62%)	27 (54%)	52 (94%)	
WAI all	29.2 (10.8)	35.6 (9.0)	26.5 (10.2)	<0.001*
WAI women	31.4 (11.1)	35.1 (8.6)	25.2 (9.7)	
WAI men	26.2 (9.8)	37.4 (11.0)	31.3 (11.2)	

*ANOVA.

The purpose of the study was to evaluate the relationship between work ability and mental fatigue using a linear regression analysis. For this, the goal to include people suffering from mental fatigue (cutoff MFS over 10) and those not, concerning work ability was achieved, even if the group without problems was smaller (Table 2). The numbers of men and women differed between groups and gender was included in the analysis. The dependent variable was work status in the workplace and the independent variables (predictors) were diagnostic group, age, gender, WAI and MFS. The result showed that the model was significant, *F*_(5,138)_ = 32.711, *p* < 0.001, and explained 53% of the variance (Adjusted R2 = 0.526). WAI and MFS were significant predictors for work status in the workplace, but neither diagnostic group, gender nor age were (Table 3).

**TABLE 3:** Linear regression analysis resulted in WAI (Work Ability Index) and MFS (Mental Fatigue Scale) as significant predictors for work status in the workplace, but this was not found for either diagnostic group, age, or gender.

	Unstandardized coefficients		Standardized coefficients	*p*-value
	B	Sd error	β	
Group	1.671	2.898	0.035	0.565
Age	0.048	0.187	0.016	0.795
Gender	2.080	5.212	0.014	0.691
MFS	0.987	0.456	0.203	0.032
WAI	3.241	0.341	0.883	<0.001

**FIGURE 1:**
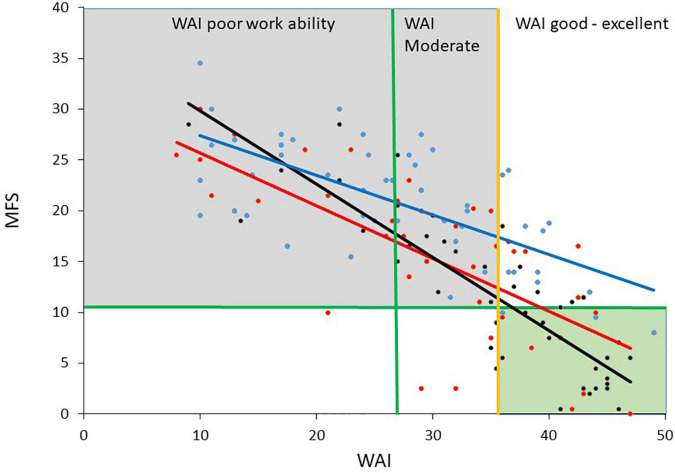
The figure shows the diagnostic groups ABI (acquired brain injury, red), HYT (hypothyroidism black) and BO (burn-out syndrome, blue), and the relationship between WAI (Work Ability Index) and MFS (Mental Fatigue Scale). The gray square indicates poor and moderate work ability according to WAI, together with mental fatigue above cutoff. The green square indicates good work ability and no significant mental fatigue.

Work Ability Index and MFS showed a high significant correlation and both scales correlated significantly with work status in the workplace and self-perceived own estimated work status (groups merged). Work status in the workplace correlated significantly with age and WAI and decreased with increasing age. Age did not correlate with MFS and self-perceived work status (Table 4). The relationship between WAI and MFS for each patient group is shown in Figure 1.

**TABLE 4:** Correlation between age, work status in the workplace, self-perceived work status in the workplace, WAI (Work Ability Index), MFS (Mental Fatigue Scale), *r*, and *p*-value.

	Age	Work status in the workplace	Self-perceived work status in the workplace	WAI	MFS
Age	x	–0.182*	–0.125	–0.185	–0.032
Work status in the workplace		x	0.710***	0.722***	−0.443***
Self-perceived work status in the workplace			x	0.840***	−0.650***
WAI				x	−0.754***

*p < 0.05, ***p < 0.001.

## Discussion

The results showed that there was a clear relationship between work ability (WAI) and mental fatigue (MFS) and both scales correlated with work status in the workplace. WAI and MFS were also significant predictors for work status in the workplace. Neither diagnostic group, age or gender, turned out to be significant predictors for work status. The connection between fatigue and work ability found here is in agreement with what is reported from other ABI, HYT and BO studies (Glise et al., 2010; Andersen et al., 2012; Waljas et al., 2014; Palm et al., 2017; Leso et al., 2020; Rutkowski et al., 2021; Western et al., 2021). This shows that mental fatigue is important to routinely screen for when work ability is assessed, specifically in patient groups where fatigue commonly occurs. This also shows the need to inform employers and the patient about the impact mental fatigue may have on return to work and to adapt working hours and working environment to what is sustainable for the patient.

It is here suggested that WAI and MFS can be used as a screening tool and this can indicate when an in-depth investigation is required. If a patient reports mental fatigue, the underlying causes should always be identified before initiation of treatment, rehabilitation and adaptation of the working environment. The intention is to avoid or minimize long-term sick leave and to improve well-being in the workplace. The patient may have several other medical disorders and/or cognitive, psychological and social problems not included in this study, which need to be evaluated in relation to mental fatigue. It is also necessary to evaluate and understand the answers from the items included in WAI and MFS. Some individuals may over-estimate their problems while others may under-estimate them due to a lack of insight, knowledge or memory problems. However, both the WAI and MFS scales have pre-defined alternatives (no likert scale), are highly consistent between and within patients and this, in turn facilitates patient follow-up.

The screening method suggested here with WAI and MFS can help health care professionals to identify people who are mentally fatigued with a reduced ability to work or return to work after illness. It has previously been shown that WAI is a sensitive screening tool to identify people on sick leave with a probable need for rehabilitation (Bethge et al., 2015) and that MFS is related to work ability (Palm et al., 2017). In this study, it was also indicated that some of the participants probably worked more than they could manage. Their self-perceived percentage of full-time working hours was lower than the level corresponding to their work status in the workplace and of those working full-time, several reported a poor to moderate work ability according to WAI. This indicates that some worked more than what they could manage. From clinical experience, several patients have a strong wish to return to ordinary life including work but they have not understood or adequately reported their reduced capacity to work. They have returned to work too early and have increased working hours too fast and worked above their limits. This resulted in total exhaustion and a need to reduce the working hours for a long time. To avoid this, early identification of patients and adaption of the working hours and working environment is important.

Among the respondents, 19 reported a second diagnosis from the background questionnaire whether they have an additional diagnosis of the three included in the study. Only one diagnosis was used for the selection of participants. The ABI was prioritized in the selection process due to the lower numbers of patients having an ABI diagnosis reported in the primary care. An additional diagnosis of those included here, as well as other diagnoses not included, could increase the burden to the perceived mental fatigue as well as working ability. As this was a survey and no additional in-depth analysis of participants was done, it was not possible to evaluate which diagnosis was the most prominent for the 19 participants with a self-reported second diagnosis, nor whether more than one diagnosis would exacerbate the mental fatigue and cause a decline in work ability. Several factors can worsen the state of people’s work ability. This highlights the need of a thorough assessment for the patient when planning for treatment and rehabilitation. In this study, the respondents only reported their total burden on mental fatigue in relation to work ability.

The design of the study was not intended to compare the diagnostic groups in terms of mental fatigue and work ability, although the results may, to some extent reflect the reality. BO was the group that reported the highest MFS and the lowest WAI ratings. BO have high impact on work ability and can cause long-term sick leave and it may take several years to return to work (Beno et al., 2021). BO is a diagnosis related to work ability and feelings of energy depletion or exhaustion while the others are medical diagnoses and are not diagnosed based on work disability and mental fatigue. The ABI group rated MFS and WAI in-between the ratings reported for BO and HYT. After an ABI, many patients are initially affected by mental fatigue to varying degrees (Andelic et al., 2020; Rutkowski et al., 2021) and recover to some extent over time (Olver, 1996). A majority with mild TBI will recover within months (Carroll et al., 2004). However, several with acquired mild injury may experience long-term problems with fatigue (Johansson et al., 2009; Winward et al., 2009). HYT was the group that reported the least problems with mental fatigue and diminished work ability. HYT can be treated with hormone replacement drugs that can have a beneficial effect on MFS and WAI, although not always (Louwerens et al., 2012).

Twenty-six percent of the people addressed responded. The number of respondents was similar between the diagnostic groups with no difference in age and education level. There was a predominance of university graduates (50–62%). By comparison, about 50% of the population in this age group in Gothenburg have a university education. There was an even gender distribution in ABI. The women were in the majority in the HTY (79%) and BO (78%) groups, and this corresponds to the gender distribution for HYT and BO in Sweden (Socialstyrelsen, 2018). The rating of MFS and WAI did not differ between men and women within the respective diagnostic groups.

### Limitations

This is a limited study with few participants. The intention was to include more participants, but only 25% responded. Nonetheless, an even number of respondents was achieved for each diagnostic group and the gender and education distribution reflect the demographics in Sweden. Studies with more participants are warranted. This study applies to Swedish employment conditions and sick-leave rules and it needs to be evaluated for use in other countries. WAI has been used internationally for 30 years and MFS for 15 years.

In conclusion, this study showed that mental fatigue is related to work ability and work status in the workplace and is present in all three diagnostic groups; ABI, HYT, and BO. WAI and MFS are suggested to be used for screening of work status in the workplace when a patient is suffering from mental fatigue and this screening can be used to indicate when an in-depth assessment is required. This screening method may also be useful for other groups of patients who commonly suffer from mental fatigue. The method can help people affected by mental fatigue to enable them to receive treatment and rehabilitation without delay and with the intention to promote a sustainable and well-functioning workplace and well-being of the individual.

## Data Availability Statement

The raw data supporting the conclusions of this article will be made available by the author, without undue reservation.

## Ethics Statement

The studies involving human participants were reviewed and approved by Etikprovningsmyndigheten. The patients/participants provided their written informed consent to participate in this study.

## Author Contributions

BJ designed the study, collected the data, analyzed the data, wrote the manuscript, and approved the submitted version.

## Conflict of Interest

The author declares that the research was conducted in the absence of any commercial or financial relationships that could be construed as a potential conflict of interest.

## Publisher’s Note

All claims expressed in this article are solely those of the authors and do not necessarily represent those of their affiliated organizations, or those of the publisher, the editors and the reviewers. Any product that may be evaluated in this article, or claim that may be made by its manufacturer, is not guaranteed or endorsed by the publisher.
